# The association between healthy beverage index and sarcopenic obesity among women with overweight and obesity: a cross-sectional study

**DOI:** 10.1186/s12902-023-01274-w

**Published:** 2023-01-31

**Authors:** Niloufar Rasaei, Rasool Ghaffarian-Ensaf, Fatemeh Gholami, Farideh Shiraseb, Alireza Khadem, Seyedeh Fatemeh Fatemi, Khadijeh Mirzaei

**Affiliations:** 1 Department of Community Nutrition, School of Nutritional Sciences and Dietetics, Tehran University of Medical Sciences (TUMS), P.O. Box:14155-6117, Tehran, Iran; 2 Department of Nutrition, Science and Research Branch, Islamic Azad University, Tehran, Iran; 3 Food Microbiology Research Center, Tehran University of Medical Sciences, Tehran, Iran

**Keywords:** Healthy beverage index, Sarcopenic obesity, Obesity and Overweight

## Abstract

**Introduction:**

Sarcopenic obesity is related to changes in body composition, loss of muscle mass, and raised adipose tissue. Beverage patterns are effective with changes in health status. Therefore, the aim of this study was to investigate the association between sarcopenic obesity (SO) and the healthy beverage index (HBI) in women with overweight and obesity.

**Methods:**

This cross-sectional study conducted on 210 overweight and obese (BMI ≥25 kg/m^2^) women aged 18–56 years. The measurement of skeletal muscle mass (SMM) and fat mass (FM) done by bioelectric impedance analyzer (BIA) (Inbody Co., Seoul, Korea) based on guidelines. The two lowest quintiles SMM and the two highest quintiles FM and body mass index (BMI) ≥30 are considered sarcopenic obesity in women. A validated and reliable semi-quantitative food-frequency questionnaire (FFQ) was used to evaluate the beverage dietary data. and RFS and NRFS was calculated. Biochemical assessments were quantified by standard approaches, and physical activity were evaluated by international physical activity questionnaire (IPAQ).

**Result:**

In this cross-sectional study, 210 overweight and obese females took part (18–56) years old). The studies were carried out using binary logistic regression. After controlling for a wide variety of confounding variables such as age, energy intake, physical activity, education, and economic status, we found a negative association between HBI and risk of SO (OR = 0.29, 95% CI = 0.35 to 1.01, *P* = 0.05).

**Conclusion:**

We observed that the odds of SO was reduced by 69% in participants with higher HBI score. More well-designed studies need to confirm our findings.

## Introduction

Sarcopenic obesity, as a major clinical problem, refers to changes in body composition including decreased skeletal muscle mass and increased fat mass (FM); this can lead to muscle dysfunction with the loss of muscle strength [1]. Sarcopenia obesity naturally occurs in older-aged adults and is related to some adverse outcomes, such as reducing the quality of life [2], raised the risk of death and disability [3, 4]. These changes in body composition usually started at 40 years of age [5]. Different methods are used to diagnose sarcopenic obesity, based on studies, the two lowest quintiles of SMM and the two highest quintiles of FM and BMI ≥30 are considered as criteria for sarcopenic obesity [5, 6]. Among the factors affecting muscle strength, nutrition plays an important role [7, 8]. Unhealthy eating habits such as food consumption of high glycemic load and sweetened beverages (SSBs) are severely affected [9].

Beverage patterns are linked to dietary and health patterns [10]. Healthy Eating Index (HEI) [11] is used to assess overall dietary patterns [12], and Healthy Beverage Index (HBI) to assess quality of individual’s nutrition and beverage intake [13]. The HBI may have important public health implications since it can be implemented as a counseling tool to improve healthy beverage selection. This index consists of eight beverage categories, total beverage energy, and fluid consumption [13], that the intake of beverages like milk, coffee, tea, and other unsweetened beverages could be different impacts on general health [14–16].

Based on some previous studies, excessive calories intake from sugary beverages, SSB, sugar, added sugars, and artificially sweetened soda is significantly associated with the risk of obesity, obesity-related diseases [17–19], lower handgrip strength, and lower skeletal muscle mass [20, 21]. While, in another study, there was no association between SSB drinkers with overweight [22]. One study showed no relation between SSB intake and percent body fat measured [23]. The frequency of SSB consumption was not associated with overweight, obesity, waist circumference (WC), or waist-to-hip ratio (WHR) [24].

According to some previous studies, coffee consumption is related to increased skeletal muscle mass, muscle strength, fat mass, and lower prevalence of sarcopenia in the middle-aged [25–27] . While, other studies the association between caffeine with muscle strength have not approved [28, 29]. Few studies have investigated the relationship between coffee consumption with the reduction of obesity risk [30, 31]. Whereas, another study reported no association between coffee consumption and change in fat-free mass index (FFMI), and was weakly associated with a reduction of body weight, fat mass and waist circumference [32]. Also The positive association between dairy products with sarcopenia [33], and the consumption of normal/high-fat milk with higher lean body and muscle mass was indicated [34].

To the best of our knowledge, the relationship between sarcopenic obesity with HBI has not been investigated so far. Consequently, based on studies conducted, the objective of the present study was to assess the association of sarcopenic obesity with HBI among adults.

## Method and materials

### Subjects

This is a cross-sectional study conducted on 210 women with overweight and obesity in Tehran. Women with a BMI ≥ 25, and aged 18–56 years were included in the study. Exclusion criteria were: cancer, history of cardiovascular diseases (CVDs), polycystic ovary syndrome (PCO), hypertension, liver disease, type 2 diabetes (T2D), kidney failure, weight loss program, stroke, thyroid disease, inflammatory disorders, taking any therapeutic drugs or supplements during the study period. The study objectives and procedures were described to the participants and a written informed consent form provided to them. The total energy consumption of women out of the range of < 500 or > 3500 kcal/day were also excluded from the study.

### Data collection

Multi stage simple random sampling was conducted on this cross-sectional study to collect 276 obese women from comprehensive health care centers of Tehran. Our population was healthy women without acute or chronic diseases. These people usually came for their annual health check-up or vaccination of their children. The inclusion criteria were following items: having 18 to 64 years of age and BMI ≥30. Having thyroid disease, kidney disease, diabetes type I and II, cardiovascular disease, menopause, any acute or chronic diseases, getting weight loss agents, pregnancy, malignancies, lactation, smoking, getting diet over the past year, receiving glucose, lipid and blood pressure lowering drugs included exclusion criteria.

Participants’ age, marital status, and level of education were record. We used a standard socio-demographic questionnaire to collect data age, education level, marital status, job, supplementation, and economic status for the Iranian population [35]. In this questionnaire, to determine the economic status, questions were asked about vehicle and house ownership, the number of family members, and having some household items. According to this questionnaire, the economic score varied from 0 to 9, and a higher score indicated a better economic situation. In the end, based on this score, the participants were classified into 3 groups: poor economic status 3–0, moderate economic status 6–3, and good economic status 7–9. The systolic and diastolic blood pressure of the left arm in a sitting position after a 5-minute resting were monitor by a hand-operated sphygmomanometer. Individual’s physical activity was assessed using the International Physical Activity Questionnaire (IPAQ) as a validated, reliable standard questionnaire [36]. Before the start of the trial, all participants signed a written informed consent form. The current study and informed consent were also accepted by the Tehran University of Medical Sciences (TUMS) local ethics committee, with the ethics number IR.TUMS.MEDICINE.REC.1399.636.

### Anthropometric measures

The height was measured at a precision of 0.5 cm with a non-stretch tape measure in a standing position and unshod of participants. Like height, hip circumference was measured by marking the most prominent part with a precision of 0.5 cm. anthropometric and clinical characteristics with BMI, weight, and WC were measured by a BIA.

### Dietary assessment

We used a validated and reliable food-frequency questionnaire (FFQ) [37] as a standard approach to assess food consumption in the form of face-to-face. This evaluation become conducted via asking individuals about the occurrence of food items consumed from an organized list of foods. The final serving amounts extracted from FFQ were convert to grams per day using home measures. We also used these data for calculating the calorie intake with standard methods. Micro and macronutrients, and energy intakes were assessed using N4 (First Data Bank, San Bruno, CA) software.

### Healthy beverage index Scoring System

In 2015, to calculate the HBI, a method was created by Duffey and Davy [13]. This method has 8 categories include diet drinks (including non-calorically sweetened coffee and tea and other artificially sweetened beverages), sugar-sweetened beverages (including fruit drinks, sweetened coffee and tea, and soda), 100% fruit juice, alcohol (including beer, wine, and liquor), full-fat milk (1.5% fat), water, unsweetened coffee and tea, and low-fat milk (1.5% fat, fat-free, and/or soy milk). The score of HBI ranges from 0 to 100. Better compliance with beverage standards is achieved by increasing the HBI scores [13]. In this study, we exclude diet drinks and alcohol with a score ranging 0 to 5 from the HBI categories, therefore, the maximum score of HBI was 90.

### Definition of sarcopenic obesity

Based on studies and definition, we defined sarcopenic obesity using the diagnostic algorithm recommended by the Asian Working Group on Sarcopenia (AWGS) performed in Asian men and women with a standard distribution of weight [5]. Two lower quintiles of SMM and two highest quintiles of FM consider as sarcopenic obesity in Iranian women. The measurement of SMM and FM done by BIA (Inbody Co., Seoul, Korea) based on guidelines.

### Statistical analysis

Kolmogorov-Smirnov test and histogram were used to evaluate the normal distribution of variables. Data related to continuous characteristics were expressed as means (SDs) and data related to categorical characteristics were expressed as numbers (%). Chi-square test was used to evaluate significant differences of categorical variables among qurtiles of HBI score and also one-way analysis of variance (ANOVA) was used to evaluate significant mean differences of continuous variables across qurtiles of HBI score cutoff points (T1: < 63, T2: 63–65, T3: 65–68, T4: 68<). The analysis of covariance (ANCOVA) was used to examine dietary intakes and general characteristics mean differences between qurtiles of HBI score after adjusted by energy intake for the dietary intakes and further with age, physical activity, and BMI for general characteristics. BMI considered as collinear variable for general characteristics. We used binary logistic regressions to assess the association between quartiles of HBI score and SO and estimate odds ratios (ORs) and 95% confidence intervals (CIs) in crude and multivariable-adjusted models. Age, energy intake, and physical activity were controlled for in the first model. Further adjustment were made for education and economic status in the second model. The first quartiles of HBI score and obese and overweight women without SO considered as reference group. In the current study, statistical analyzes via Statistical Package for Social Sciences (version 26; SPSS Inc., Chicago, IL, USA) was performed. *P*-value< 0.05 was considered statistically significant and 0.05, 0.06, and 0.07 was considered marginally significant.

## Results

### Study population characteristics

A total of 210 women were included in the statistical analysis. The prevalence of participants with sarcopenic obesity in this study was 7.1%. The HBI has a mean and standard deviation (SD) of 65.12 ± 4.40 in this study. The mean (SD) of age, weight, BMI, WC, and body fat mass of individuals were 36.18 ± 8.44 years, 80.42 ± 12.00 kg, 30.79 ± 4.17 kg/m2, 98.70 ± 9.77 cm, and 33.42 ± 8.11 kg respectively. The mean and SD of SBP and DBP were 112.49 ± 13.21 and 78.54 ± 10.01 respectively.

This cross-sectional study looked at socioeconomic status, such as marriage, occupation, education, and economic status. According to the findings, 202 (96.2%) of the participants were employed, and 153 (72.9%) of the participants were married. The majority of those who took part possessed a diploma (76 (36.2%)) or a bachelor’s degree or higher (101 (48.1%)). In this survey, 54 people (25.7%) said they were in good financial shape.

### The differences in means of body composition and blood pressure across HBI

Table 1 shows the characteristics of the participants with respect to the quartiles of the HBI. There was no statistically significant association between any of the variables and the HBI (Table 1).

**Table 1:** Mean and SD of general characteristics according to quartiles of healthy beverage index in obese and overweight women (*n* = 210)

Variables	HBI				***P***-value†	***P***-value*
	Q1 (< 63) ***N*** = 75	Q2 (63–65) ***N*** = 43	Q3 (65–68) ***N*** = 47	Q4 (> 68) ***N*** = 45		
Age (years)	35.72 ± 8.35	34.79 ± 8.59	39.04 ± 8.05	35.41 ± 8.44	**0.07**	0.17
Body weight (Kg)	80.42 ± 11.16	79.36 ± 12.57	79.25 ± 13.69	82.77 ± 10.90	0.49	0.14
Height (cm)	161.90 ± 5.60	160.68 ± 5.74	160.79 ± 6.18	162.33 ± 5.85	0.43	0.17
BMI (Kg/m^2^)	30.66 ± 3.93	30.85 ± 4.26	30.61 ± 4.69	31.19 ± 4.00	0.91	0.43
IPAQ (MET min-week)	800.49 ± 840.00	1299.90 ± 1448.44	839.14 ± 590.29	1354.44 ± 1504.76	**0.03**	0.12
**Body composition**						
WC (cm)	98.09 ± 9.45	98.47 ± 10.27	97.98 ± 10.85	100.82 ± 8.44	0.47	0.19
HC (cm)	113.91 ± 9.73	112.40 ± 8.02	114.17 ± 11.22	115.21 ± 8.71	0.63	0.63
WHR	0.92 ± 0.05	0.93 ± 0.05	0.93 ± 0.05	0.94 ± 0.04	0.19	0.08
BFM (kg)	33.03 ± 7.99	33.19 ± 8.29	33.47 ± 9.19	34.29 ± 6.95	0.87	0.65
BF (%)	40.67 ± 5.58	41.02 ± 5.26	41.76 ± 5.10	40.83 ± 5.80	0.74	0.72
SMM (kg)	26.02 ± 3.32	25.51 ± 3.76	25.06 ± 3.52	26.21 ± 2.91	0.33	0.13
FFM (%)	47.39 ± 5.59	46.47 ± 6.28	45.73 ± 6.01	47.67 ± 4.88	0.31	0.10
FMI	12.67 ± 3.13	12.87 ± 3.15	12.97 ± 3.56	13.29 ± 3.14	0.80	0.68
FFMI	18.04 ± 1.51	17.97 ± 1.62	17.64 ± 1.54	21.20 ± 20.06	0.22	0.54
**Blood pressure**						
SBP (mmHg)	113.72 ± 11.47	108.82 ± 13.27	113.44 ± 13.93	112.92 ± 15.02	0.25	0.31
DBP (mmHg)	80.29 ± 8.44	76.09 ± 10.86	79.04 ± 11.95	77.29 ± 9.16	0.14	0.39
**Education category**						
Illiterate	0.0 (0)	66.7 (2)	0.0 (0)	33.3 (1)	0.19	0.01
Under diploma	17.9 (5)	32.1 (9)	25.0 (7)	25.0 (7)		
Diploma	35.5 (27)	19.7 (15)	26.3 (20)	18.4 (14)		
Bachelor and higher	41.6 (42)	16.8 (17)	20.8 (21)	20.8 (21)		
**Marital status**						
Single	34.5 (19)	25.5 (14)	18.2 (10)	21.8 (12)	0.65	0.34
Married	35.9 (55)	19.0 (29)	24.8 (38)	20.3 (31)		
**Supplement intake**						
Yes	51.2 (43)	25.0 (21)	20.2 (17)	3.6 (3)	0.76	0.42
No	42.2 (27)	29.7 (19)	23.4 (15)	4.7 (3)		
**Job category**						
Employed	36.6 (74)	20.8 (42)	22.3 (45)	20.3 (41)	0.65	0.69
Unemployed	50.0 (1)	0.0 (0)	50.0 (1)	0.0 (0)		
**Economic status**						
Poor	50.0 (28)	17.9 (10)	14.3 (8)	17.9 (10)	0.27	0.05
Moderate	34.1 (28)	24.4 (20)	25.6 (21)	15.9 (13)		
Good	31.5 (17)	20.4 (11)	22.2 (12)	25.9 (14)		

*SD* Standard deviation, *HBI* Healthy beverage index, *BMI* Body mass index, *IPAQ* International physical activity questionnaire, *WC* Waist circumference, *HC* Hip Circumference, *WHR* Waist height ratio, *SMM* Skeletal muscle mass, *FFM* Fat-Free Mass, *FMI* Fat Mass Index, *FFMI* Fat-Free Mass Index, *BFM* Body fat mass, *BF* Body fat, *SBP* Systolic blood pressure, *DBP* Diastolic Blood Pressure

† Calculated by analysis of variance (ANOVA)

* Analysis of covariance (ANCOVA) was performed to adjust potential confounding factors (age, BMI, energy intake, physical activity)

BMI considers as the collinear variable for anthropometrics and body composition variables

Values are represented as means (SD)

Categorical variables: %(N)

*p*-values < 0.05 were considered as significant

*p*-values < 0.06 and 0.07 were considered as marginally significant

### Comparison of dietary intake in participants across HBI

The nutritional and food group intakes of participants across HBI quartiles are shown in Table 2. Participants in the highest category of HBI had greater intakes of potassium (*P* = 0.02), vitamin B6 (*P* = 0.005), whole grains (*P* = 0.004), and fruits (*P* = 0.02) after adjusting for calorie intake. The lowest quartile of HBI patients had increased vitamin E intake (*P* = 0.01). The mean difference in MUFA and linolenic acid became statistically significant (*P* = 0.05) (Table 2).

**Table 2:** Nutrient intake according to quartiles of healthy beverage index in obese and overweight women (*n* = 210)

Variables	HBI					
	Q1 (< 63) ***N*** = 75	Q2 (63–65) ***N*** = 44	Q3 (65–68) ***N*** = 45	Q4(> 68) ***N*** = 43	***P***-value†	***P***-value^*^
**Dietary intake**						
Energy (kcal/d)	2525.68 ± 670.80	2876.70 ± 765.43	2353.98 ± 772.66	2713.56 ± 811.41	**0.008**	–
Protein (g/d)	86.08 ± 25.73	96.63 ± 29.26	84.90 ± 29.61	91.75 ± 29.93	0.16	0.49
Carbohydrate (g/d)	355.75 ± 116.08	408.13 ± 119.20	327.14 ± 123.75	403.24 ± 124.93	**0.004**	0.08
Total fat (g/d)	92.16 ± 31.28	104.87 ± 36.02	86.00 ± 28.37	91.22 ± 33.59	**0.05**	0.16
Cholesterol (mg/d)	238.35 ± 97.77	273.23 ± 128.41	256.20 ± 92.57	270.26 ± 97.71	0.25	0.25
SFA (mg/d)	26.65 ± 9.82	31.58 ± 13.67	27.21 ± 10.69	26.99 ± 11.22	0.12	0.08
MUFA (g/d)	31.83 ± 12.15	33.60 ± 11.59	27.87 ± 9.97	29.45 ± 10.84	0.08	**0.04**
PUFA (g/d)	20.50 ± 9.65	21.65 ± 8.71	17.74 ± 7.16	18.34 ± 8.73	0.12	0.11
Linolenic acid (g/d)	1.04 ± .59	1.59 ± .60	1.31 ± .68	1.08 ± .52	**0.001**	**< 0.001**
Linoleic acid (g/d)	18.05 ± 9.33	18.47 ± 8.24	14.89 ± 6.52	15.65 ± 8.18	0.09	**0.07**
Sodium (mg/d)	4195.75 ± 1335.16	4672.32 ± 1460.87	3781.65 ± 1311.84	4231.22 ± 1382.43	**0.03**	0.57
Potassium (mg/d)	4110.79 ± 1566.16	4622.94 ± 1476.27	4130.37 ± 1459.413	4895.02 ± 1666.32	**0.03**	**0.05**
Vitamin D (μg/d)	2.04 ± 1.62	1.84 ± 1.51	1.81 ± 1.08	2.25 ± 1.76	0.53	0.54
Vitamin E (mg/d)	19.35 ± 11.07	16.13 ± 6.21	13.84 ± 5.86	16.16 ± 9.60	**0.01**	**0.001**
Thiamin (μg/d)	2.04 ± 63	2.27 ± .61	1.81 ± .61	2.11 ± .72	**0.01**	0.57
Riboflavin (mg/d)	2.15 ± .87	2.37 ± .78	2.06 ± .62	2.39 ± 1.02	0.16	0.80
Niacin (mg/d)	24.01 ± 7.33	27.74 ± 8.97	23.14 ± 9.60	26.89 ± 10.93	**0.04**	0.74
Vitamin B6 (μg/d)	2.03 ± .64	2.31 ± .76	2.08 ± .72	2.39 ± .75	**0.03**	**0.02**
folate (μg/d)	588.44 ± 177.88	661.77 ± 162.17	535.26 ± 158.59	652.83 ± 184.06	**0.002**	0.13
Vitamin B12 (μg/d)	4.16 ± 1.91	4.89 ± 2.72	4.21 ± 1.94	5.03 ± 3.72	0.20	0.67
Vitamin K (μg/d)	185.35 ± 108.12	263.82 ± 251.16	213.37 ± 135.23	274.43 ± 360.64	0.11	0.33
Phosphorus (mg/d)	1609.31 ± 497.78	1776.36 ± 533.26	1546.51 ± 509.85	1695.16 ± 574.44	0.18	0.83
Vitamin C (μmol/L)	179.01 ± 159.25	197.64 ± 103.51	181.16 ± 106.79	179.01 ± 159.25	0.16	0.18
Calcium (mg/d)	1138.60 ± 416.045	1251.28 ± 438.75	1110.86 ± 369.98	1198.90 ± 475.11	0.40	0.87
Magnesium (mg/d)	448.85 ± 145.78	507.42 ± 145.84	425.03 ± 138.47	497.24 ± 168.06	**0.02**	0.76
Zinc (mg/d)	12.80 ± 4.29	14.19 ± 4.19	12.03 ± 3.94	13.69 ± 4.83	0.09	0.95
Total fiber (g/d)	42.75 ± 19.13	51.77 ± 15.83	38.68 ± 15.15	47.39 ± 20.12	**0.005**	0.01
**Food groups**						
Grains (g/d)	458.89 ± 239.94	486.59 ± 200.30	360.28 ± 172.86	477.09 ± 302.97	**0.04**	**0.04**
Fruits (g/d)	477.82 ± 372.87	541.93 ± 312.01	475.80 ± 325.50	671.42 ± 392.53	**0.02**	**0.04**
Vegetables (g/d)	418.82260.28	472.20 ± 216.73	431.50 ± 273.45	541.80 ± 342.54	0.12	0.24
Nuts (g/d)	13.02 ± 16.76	19.47 ± 19.20	16.14 ± 13.71	16.01 ± 20.87	0.30	0.44
Legumes (g/d)	53.80 ± 44.74	60.71 ± 40.04	49.08 ± 41.89	56.35 ± 43.37	0.64	0.92
Dairy (ml/d)	391.09 ± 244.07	379.21 ± 223.02	377.00 ± 194.55	439.59 ± 353.89	0.65	0.41
Meat (g/d)	56.54 ± 38.99	73.73 ± 60.40	70.08 ± 59.34	70.94 ± 41.72	0.21	0.22
Processed food (g/d)	23.19 ± 26.73	19.73 ± 15.61	26.33 ± 30.93	19.62 ± 19.78	0.53	0.16

*MUFA* Monounsaturated fatty acid, *PUFA* Polyunsaturated fatty acid, *SFA* Saturated Fatty Acid

Values are represented as means ±SD

† Calculated by analysis of variance (ANOVA)

*ANCOVA (*P*-value*) was performed to adjust potential confounding factors (energy intake)

*p*-values < 0.05 were considered as significant

### Association of the HBI with sarcopenic obesity

Using binary logistic regression, we looked at the relationship between adherence to the HBI and SO in crude and adjusted models. There was no link between quartiles of HBI and SO in the crude model (OR = 1.06, 95% CI = 0.24 to 4.68, *P* = 0.93), but we discovered a negative association between quartile3 of HBI and the odds of SO (OR = 0.29, 95% CI = 0.35 to 1.01, *P* = 0.05) after adjusting for relevant variables (age, energy intake, physical activity, education, and economic position) and the odds of SO was reduced by 69% in Q3 compare to Q1 (Table 3). P-trend was not significant in any of the models.

**Table 3:** Odds ratio and 95% confidence interval for the association between quartiles of healthy beverage index and sarcopenic obesity in obese and overweight women (*n* = 210)^a^

	HBI							
	Q1^b^	Q2	***p***-value	Q3	***p***-value	Q4	***p***-value	***p***-trend
Crude	1.00	0.65 (0.12 to 3.54)	0.62	1.60 (0.43 to 5.86)	0.47	1.06 (0.24 to 4.68)	0.93	0.67
Model 1	1.00	0.39 (0.03 to 4.16)	0.43	0.61 (0.79 to 1.97)	0.18	0.57 (0.06 to 5.25)	0.62	0.87
Model 2	1.00	0.56 (0.03 to 4.05)	0.09	0.29 (0.35 to 1.01)	**0.05**	1.12 (0.09 to 7.94)	0.92	0.71

^a^Based on binary regression model in crude and adjustment models

^b^Quartile 1 considered as reference group

Obese and overweight women without SO consider as reference group

Model 1: Adjusted for age, energy intake, physical activity

Model 2: Adjusted for age, energy intake, physical activity, education, economic status

*p*-values < 0.05 were considered as significant and 0.05, 0.06, and 0.07 was considered marginally significant

## Discussion

The prevalence of overweight and obesity have been rapidly increased in the world and this cross-sectional study aimed to reveal the association between HBI with SO among women with overweight and obesity. Our results showed that there is a statistically significant association between HBI and SO among participants. The odds of SO were reduced by 69% in participants with higher HBI score. We investigated the relation of HBI with SO for the first time and no studies have been done in this field.

Based on our results, participants with higher HBI score, had lower risk of SO. For some studies the association of SO with moderate alcohol drinking was clear [38], and for some studies this association was unclear and they didn’t observe any link between alcohol consumption and SO and even observed that drinking alcohol had protective effect of sarcopenia [39]. One potential mechanism for effect of alcohol on SO could be the inhibition function of ethanol on protein synthesis in muscles and in opposite increasing autophagy of muscles [40]. Autophagy is a procedure that essential for renewal of mitochondria and effective for maintenance of muscle mass [41].

In some studies, it revealed that consumption of coffee was associated with lower risk of sarcopenia [27, 41]. Compare to 1 cup of coffee per day, consumption 3 or more cup of coffee daily decreased the risk of SO about 60% [41]. In another study, the risk of sarcopenia reduced with drinking 1 cup of coffee per day [27]. In a Korean study, it was reveal that coffee consumption reduced the risk of sarcopenia in women and it had no effect of men [42]. Duo to various stresses on muscles, the rate of autophagy increased [43] and according to studies, chronic consumption of coffee can stimulated autophagy [44]. Furthermore, coffee have antioxidant components like polyphenols that induce autophagy [27, 45]. Because of antioxidant properties of coffee, it could reduce the risk of SO [41]. Like coffee, tea consumption has beneficial effect of SO because of its antioxidant properties [46, 47].

According to studies, consumption of fruit juices could reduce SO [48, 49]. By reduction of oxidative stress duo to antioxidants components of fruits such as vitamin C and carotenoids [50], the risk of SO decreased [51]. Vitamin C as a water-soluble vitamin has important effect on sarcopenic risk factors of skeletal muscle and it is known that fruit juices is one of the greatest contribution of vitamin C intake [52]. Vitamin C can help to the synthesis of carnitine and collagen in skeletal muscle [53–56]. Another mechanism by which fruit juices could have beneficial effects on SO is reducing proteolysis and amino acid catabolism [57, 58]. Furthermore, alkaline-forming properties of fruits make its function like a buffer to reduce the risk of SO by decreasing oxidative stress and increasing muscle mass [59].

In terms of milk, it has been reveal that milk and dairy products help to maintain muscle mass and reduce the risk of SO [60]. In one study, it was showed that consumption of milks in men and women was lower in sarcopenia group and it was notably that proper consumption of milk in women could reduce sarcopenia ratio [61]. According to a study, milk consumption might lead to improving muscle mass [62] and prevent SO [63]. Milk contains some anti-oxidative components like β-lactoglobulin that have positive effects on SO [64].

Sugar-sweetened beverages (SSBs) consumption might lead to impaired glucose, lipid metabolism, reduce protein synthesis, and less efficient muscle contraction that are all might lead to sarcopenia [65–68]. Consumption added sugar foods like SSBs by adults put them in the situation that achieve inadequate dietary proteins intake, therefore, their muscle mass went to loss and they prone to SO [69, 70].

The strength of this study includes following items: our sample size was collected from same location in same period, a reliable and validated FFQ were used to evaluate the intakes of participants, and we used a standard and validated questionnaire (IPAQ) to evaluate the physical activity of participants. Despite of strengths, the limitation of this study includes following items: the study population of our study is small and it is important that in the future study authors use bigger sample size, this was a cross-sectional study and for this reason we cannot investigate the causality, we investigated the association of HBI with SO just on women with overweight and obesity and for this reason the results of this study cannot generalized to all people.

## Data Availability

The data that support the findings of this study are available from correspond author but restrictions apply to the availability of these data, which were used under license for the current study, and so are not publicly available. Data are however available from the authors upon reasonable request and with permission of correspond author.
